# The Value of Multi-targeted Fecal DNA Methylation Detection for Colorectal Cancer Screening in a Chinese Population

**DOI:** 10.7150/jca.47214

**Published:** 2021-01-15

**Authors:** Heiying Jin, Jun Wang, Chunxia Zhang

**Affiliations:** Department of colorectal surgery, The Second Affiliated Hospital of Nanjing University of Chinese Medicine, 23 Nanhu Road, Nanjing 210017, China.

**Keywords:** colorectal cancer, colorectal polyps, screening, fecal DNA methylation

## Abstract

**Objective:** To design a multi-targeted fecal DNA methylation kit and explore its value for clinical application among Chinese people.

**Methods:** Based on previous research, a multi-targeted fecal DNA methylation detection kit, using four genes, was designed and clinically validated.

**Results:** The methylation PCR from 279 patients met the requirements for the detection criteria. When all four molecular markers were negative, the negative predictive value (NPV) for colorectal cancer was 100% and the NPV for colorectal polyps was 84.21%.

When one molecular marker was positive, the sensitivity (Se) for colorectal cancer was 76.4%-90.3%, the specificity (Sp) was 68.3-93.4%, and the positive predictive value (PPV) for colorectal cancer was 54.5-85.5%, and the NPV was 87.0-95.0%. For colorectal polyps, the Se was 41.0-52.5%, Sp 69.5-91.5%, and the PPV for colorectal polyps was 41.0-70.3%, the NPV was 75.2-79.3%.

When two molecular markers were positive, the Se for colorectal cancer was 52.6-73.7%, the Sp was 93.2-98.3%, the PPV for colorectal cancer was 84.6-96.2%, the NPV was 76.0-85.3%. For colorectal polyps, the Se was 25.9-40.7%, Sp was 93.2-98.3%, PPV for screening of colorectal polyps was 63.6-90.0%, and the NPV was 73.3-78.1%.

When three molecular markers were positive, the Se for colorectal cancer was 31.6-52.6%, the Sp was 98.3-100.0%, the PPV for colorectal cancer was 94.4-100.0%, the NPV was 73.4-76.6%. For colorectal polyps, the Se was 14.8-25.9%, and Sp was 98.3-100.0%, the PPV for colorectal polyps was 85.7-100.0%, the NPV was 72.0-74.7%.

When four molecular markers were positive, the Se for colorectal cancer was 31.6%, the Sp was 100.0%, and the colorectal cancer PPV was 100.0% and the NPV was 69.4%. For polyps, the Se was 14.8%, Sp was 100.0%, and PPV was 100.0% and the NPV was 72.0%.

**Conclusion:** The multi-targeted fecal DNA methylation detection kit for colorectal cancer and polyps had the sensitivity and specificity to meet the requirements for screening of colorectal tumors, which is easy to operate, has stable results and important clinical value. Among the four molecular markers studied, when one marker was positive for DNA methylation, colonoscopy was required; as the number of positive methylation markers increased, the specificity for the diagnosis gradually increased as well.

## Introduction

Colorectal cancer is preventable and curable. However, the incidence and mortality associated with colorectal cancer has not decreased significantly. Tumor stage is still an independent risk factor associated with colorectal cancer. The most effective way to prevent colorectal cancer and improve clinical outcomes is the detection of colorectal polyps and early stage colorectal cancer 1-3. Fecal occult blood testing (FOBT) is the most widely used method for colorectal cancer screening. However, due to poor specificity of guaiac-based FOBT, many patients have to repeatedly undergo "negative" colonoscopy exams 4-5. Immunochemical FOBT (iFOBT) were developed with improved sensitivity and specificity 6. However, studies showed that the sensitivity of iFOBT was imperfect and some colorectal tumor may be missed. Wakamura et al. 7 studied 919 subclinical patients, 276 cases were iFOBT positive and 643 cases were iFOBT negative. They found in iFOBT negative groups, there exist 49.3% (318/643) cases with 513 colorectal neoplasm lesion. Among of them, 6.1% were advanced adenoma, 0.16% were colorectal cancer and 40.4% were Non-advanced neoplasia. This study implied that have high false negative. Khuhaprema et al. 8 detected iFOBT in over 80000 people, 873 (1.1%) out of them were found positive. To date 627 (72.0%) iFOBT-positive persons have had colonoscopy in which 3.7% had CRC and 30.6% had adenomas. From the perspective of colorectal cancer screening, 65.7% of patients underwent negative colonoscopy.

In theory, FOBT can only be detected by bleeding in the intestine. Although iFOBT can detect bleeding as low as 100ng/ml 6, the tumor without bleeding or small amount of bleeding may not be detected.

The gut mucosa is regularly renewed; cells from this mucosa are shed with stool. Screening for colorectal tumors, using DNA molecular markers on the mucosa that is present with stool, might be an effective way to screen for colorectal cancer. Imperiale et al. 9 reported on a fecal DNA methylation test to screen for colorectal cancer. Studies have shown that the sensitivity for colorectal cancer is 92%, the sensitivity for colorectal polyps is 42%, and the specificity is over 90%. Such findings indicate that fecal DNA methylation can be used as a valuable tool for screening of colorectal cancer and adenomas. However, DNA methylation is related to human racial groups, dietary habits, and the environment. Therefore, there may exist differences in the DNA markers used in Chinese populations when compared to Western populations 10. In previous research, our team investigated a series of DNA methylation markers and studied their screening value for the detection of colorectal tumors. Our study showed that SNCA, SPG20, Septin9, and FBN1 can be used for colorectal cancer screening 11-12. Our team designed a screening system that included the four molecular markers: SNCA, SPG20, Septin9, and FBN1. We established positive and negative internal control systems to guarantee the accuracy of screening for colorectal tumors. This study was performed to validate the clinical value of fecal colorectal tumor screening using these four DNA markers in a Chinese population.

## Materials and Methods

### 1. DNA makers and negative and positive internal control

In this study, SNCA, SPG20, Septin9, and FBN1 were selected as molecular targets 11-12. A methylated DNA was used as a positive control and a non-methylated DNA segment as a negative control. ACTB (β-actin) was used as an internal control. By detecting the amplification of control genes, we tested whether the system was effective.

PCR primers were designed for the regions of Septin9, SNCA, SPG20, and FBN1 without CpG double bases as in our previous reports 11-13. We designed blockers in the sequence regions without methylation, to make the methylation sequence preferentially amplify after it was transformed using sulfite.

### 2. Clinical cases and methylation-specific PCR detection

#### 2.1 Case Information

279 cases were enrolled in this study. Sixty-two patients with colon cancer, 71 cases with colon adenoma and 146 patients with a normal colonoscopy at the Second Affiliated Hospital of Nanjing University of Traditional Chinese Medicine were recruited into the study from May 2016 to May 2020. All patients signed the informed consent, and the study obtained Approval by the Ethics Committee of the Second Affiliated Hospital of Nanjing University of Traditional Chinese Medicine (KY2014018). Participants' clinical data are shown in Table 1.

#### 2.2 methylation-specific PCR detection

All stool samples were handled in a blinded fashion during storage and processing. DNA extraction and qMSP analysis were performed. Patient samples were collected and processed according to the method previously published by our group 9-11. Briefly, the collection was as follows: collect, one-time, a full stool sample (25-100g), collects intestinal mucosa with an automatic intestinal mucosal collector 9, and then extract intestinal mucosal DNA, and transform the DNA with sulfite. Perform fluorescent PCR detection and read the results to calculate the Ct value.

#### 2.3 Result Judgment and Interpretation

**Figure 1:**
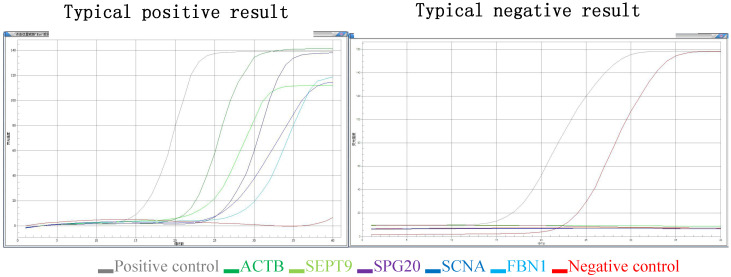
** Typical positive results and typical negative results.** Different color lines represent different molecular markers in the graph. The gray line represents positive control, the red line represents negative control, the deep green line represents ACTB, the light green line represents SEPT9, the brown line represents SPG20, the deep blue line represents SNCA and the light blue line represents the FBN1.

(1) Evaluation of experiment quality: If there was no amplification or Ct value in the detection channel of the negative control, the analysis was continued; otherwise, the experiment was considered invalid and had to be repeated.

(2) Evaluation of the positive and negative control gene detection: If the positive control channel had amplification and the Ct value was ≤ 25, the analysis was continued. If the positive control channel had a large Ct value or no amplification, but the mutation site was detected and amplified and the Ct value was ≤ 38, the analysis was continued. If the positive control channel had a large Ct value or no amplification, and the mutation site was detected without amplification or there was amplification but the Ct value was > 38, the analysis could not be continued; this indicated that the experiment had failed, and needed to be repeated. If the negative control gene control channel showed amplification, this implied that the experiment had false positive results and had to be repeated.

(3) Judgment of gene mutation in the DNA sample to be tested: if the detection channel of a mutation site in the sample was amplified and the Ct value was ≤ 35, the mutation result of the sample was considered positive. If the Ct value was > 38, or no amplification was noted, then the mutation result of the sample was determined to be negative. If 35 < Ct value ≤ 38, the experiment needed to be repeated. If the Ct value was still within this range, the sample mutation result was suspected to be positive (the Ct value may fluctuate due to low mutation content) (Figure 1).

### 3. Statistical analysis

The data was processed with SPSS 18.0. The count data was tested by the Chi-square test or Fisher's exact test. The measurement data was expressed by mean ± SD and the t-test was used. Receiver operating characteristic (ROC) analysis was used for the marker gene diagnosis Value; The sensitivity, specificity, positive predictive value, and negative predictive value of each index was calculated using the results of colonoscopy as the “gold standard”.

## Results

### 1. Relationship between the methylation of SNCA, SPG20, FBN1 and SEPT9 and clinicopathological characteristics

Detection of methylation in all 279 patients met the criteria requirements and the results could be analyzed. The methylation rates of SNCA in colorectal cancer, polyps and normal mucosa were 90.32%, 46.47% and 14.38% respectively. The methylation rates of SPG20 were 85.48%, 56.33% and 27.40% respectively. The methylation rates of FBN1 were 79.46%, 50.70% and 13.01% respectively. The methylation rates of Septin9 were 91.93%, 56.33% and 6.16% respectively. The stages were divided into groups I-II, III-IV and polyps' sizes were <0.5cm, 0.5-1cm and ≥1cm. The polyps were divided into tubular, tubulovillous and villous according to the pathological features. The p-values of the methylation among different targets in colorectal cancer, polyps and normal mucosa were included in Table 2.

### 2. The sensitivity (Se), specificity (Sp), positive predictive value (PPV) and negative predictive value (NPV) of different molecular marker combinations for colorectal cancer and adenoma screening

Using the colonoscopy results as the gold standard, we calculated Se, Sp, PPV and NPV of one, two, three, or four molecular markers with methylation positive for colorectal cancer, colorectal adenomas, and colorectal cancer and adenomas. When all four molecular markers were negative, the NPV for colorectal cancer was 100%, and the NPV for colorectal adenomas was 84.21%.

#### 2.1 Se, Sp, PPV and NPV of colorectal cancer and adenoma screening when one molecular marker was positive for DNA methylation

When one molecular marker was positive for DNA methylation, the methylation rate for four molecular markers, in colorectal cancer and adenoma, was higher than that of patients without these findings, and the difference was statistically significant. The Se, Sp, PPV and NPV of one molecular marker positive for colorectal cancer and adenoma screening are shown in Table 3.

#### 2.2 Se, Sp, PPV and NPV of colorectal cancer and adenoma screening when two molecular markers were positive for DNA methylation

When two molecular markers were positive for DNA methylation, the difference between cancer and adenomas was significantly different from normal mucosa. The specificity of screening for cancer or polyps was significantly increased, reaching more than 93%; however, in such cases the sensitivity decreases. The Se of cancer screening was 52.6% -73.7%, and the Se of adenoma screening was 25.9-40.7%, with PPV increasing and NPV decreasing. Table 4 shows the Se, Sp, PPV and NPV of screening for colorectal cancer and adenomas when two molecular markers were positive.

#### 2.3 Se, Sp, PPV and NPV of colorectal cancer and adenoma screening when 3 or 4 molecular markers were positive for DNA methylation

When 3 or 4 molecular markers were positive for DNA methylation, the differences between cancer and adenoma was significant when compared to normal mucosa. The specificity of screening for cancer or adenomas was close to 100%, but the sensitivity decreased. The sensitivity for cancer screening was 31.6-52.6%, and the sensitivity for adenoma screening was 14.8-25.9%. The PPV increases and the NPV decreases. Table 4 shows the Se, Sp, PPV and NPV of screening for colorectal cancer and adenoma when 3 or 4 molecular markers were positive (Table 5).

## Discussion

iFOBT had a higher sensitivity and specificity in colorectal cancer screening and plays an important roles in detection of early stage colorectal cancer, theoretically, iFOBT can only find the blood in the intestinal cavity. Any diseases with intestinal bleeding such as enteritis, diverticulitis, inflammation bowel disease (IBD) and so on, may produce false positive; at the same time, if the colon neoplasm does not bleed, it will appear false negative. Table 6 summarizes the studies on screening for colorectal cancer and adenoma by iFOBT in recent years. The sensitivity of colorectal cancer and adenoma screening is not ideal. The meta-analyses showed that the sensitivity of colorectal adenoma was as low as 28%. iFOBT can only find the blood in the intestinal cavity. Any diseases with intestional bleeding such as enteritis, diverticulitis, inflammation bowel disease (IBD) and so on may produce false positive; at the same time, if the colon neoplasm does not bleed, it will appear false negative. Therefore, a supplementary of iFOBT should be developed 21-22.

The detection of tumor molecular markers in stool is a valuable screening method for colorectal cancer. It is also easily accepted by patients because of its non-invasiveness and low cost 23-24. There are significant differences in the molecular findings of a tumor and normal tissues. Colorectal mucosa can fall off and be present in the feces during development of colorectal cancer, which provides the possibility for the detection of molecular alteration in feces for screening of colorectal cancer and adenomas 25-26. Studies have shown that stool molecular markers that can be used for CRC screening mainly include four types: CpG island methylation, microsatellite instability, long-chain DNA and gene mutations. Because gene methylation markers have the characteristics of strong stability, easy detection, high sensitivity, and strong specificity, the detection of methylation of genes in feces is an important method as a molecular marker for CRC screening 27-28. However, DNA methylation has racial differences, is affected by diet and the environment. Therefore, there would be expected to be differences in DNA markers in Chinese and Western populations 11-12. The molecular targets that are highly sensitive in Western populations are not sensitive in Chinese populations. Therefore, molecular markers with high sensitivity for colorectal cancer screening in Chinese people are needed. In our previous research, we found that the DNA methylation rate of four molecular markers SNCA, SPG20, FBN1 and septin-9 had significant differences when colorectal cancer, colorectal adenoma and normal mucosa were compared. Therefore, they are suitable for colorectal cancer screening 11-13.

We designed a multi-targeted DNA methylation screening kit for colorectal tumors. We used positive and negative methylation controls to perform quality control on the reaction system, thereby eliminating false positives and false negatives and increasing the accuracy of screening. In this study, the test results of all 279 patients, the positive controls and the negative controls were in line with the design, suggesting that the results of this group of studies were reliable.

For the specificity of detection, we used four targets for detection at the same time. When all four molecular marker DNA methylation tests were positive, whether it was an adenoma, cancer or cancer plus adenoma, the specificity of diagnosis was 100%, and the positive predictive value is 100%. In other words, if all four signs were positive, the patient was thought to have colorectal cancer and/or adenoma; colonoscopy and treatment followed. Using the Cologuard kit, the sensitivity for colorectal cancer was 92.3%, and the sensitivity for colorectal polyps was 42.4%; the specificity was 86.6% 10.

In our study, when three out of four markers were positive, the specificity was more than 98.3%, and the positive predictive value was more than 85.7%; which means that when performing colonoscopy, a "negative" colonoscopy would be predicted in 15% of cases or less. The efficiency of colonoscopy was significantly improved. When two molecular markers were positive, the specificity was more than 93.2%. Except that the PPV for SNCA + SPG20 and SNCA + FBN1 were slightly lower, the PPVs of other combinations reached more than 80%. If a colonoscopy was performed, the positive rate was high. When one markers was positive, the specificity for colorectal cancer ranged from 68% to 93%, the specificity for colorectal adenoma was ranged from 77% to 99%. Especially when the FBN1 and SEPT9 were positive, the specificity were higher. The PPV of a colorectal tumor (cancer+adenoma) was more than 71%. If a colonoscopy was performed in these patients, only 30% of the examinations were "negative". SPG20 was with lower specificity, we will optimize the molecular markers according to the study.

From a sensitivity perspective, the more markers applied, the lower the sensitivity. For example, when all four markers were positive, the sensitivity for colorectal cancer was only 31.6%, and the sensitivity for colorectal polyps was only 14.8%. When two or three markers were positive for DNA methylation, the sensitivity for colorectal cancer and adenoma was only 24.6%. However, when one molecular marker was used for DNA methylation, the sensitivity increased. When a single molecular marker was positive, the sensitivity for colorectal cancer was 76.4% to 90.3%, the sensitivity for colorectal adenoma was 41.0% to 52.5%, and the sensitivity of colorectal cancer and adenoma was 72.6% to 99.2%, which is ideal for molecular markers. Therefore, in terms of sensitivity, as long as one of the four markers was positive and the positive predictive value of colorectal cancer and adenoma was more than 71%, the recommendation was for the patient to have a colonoscopy.

When all four molecular markers were negative, the NPV for colorectal cancer was 100%, and the NPV for colorectal polyps was 82.8%. That is, when all four molecular markers were negative, the test subject had a risk for colorectal cancer of zero, and the risk of colorectal polyps was 17.2%. They don't need a colonoscopy for colorectal cancer screening.

In summary, this study confirmed that a multi-targeted fecal DNA methylation screening kit has the sensitivity and specificity to meet the requirements of screening for colorectal cancer. It is easy to perform, has stable results, and important clinical value. When one of four markers was positive for DNA methylation, colonoscopy was recommended. As the number of methylation markers increased, the specificity of diagnosis gradually increased. When more than two markers were positive for DNA methylation, the patient's PPV was over 90%, which indicated a very high risk of colorectal cancer or adenoma. Colonoscopy and treatment were needed immediately to detect colorectal cancer or adenoma. When all four molecular markers were negative, the possibility of colorectal cancer was very low.

## Figures and Tables

**Table 1:** Clinicopathological characteristics of cases (n=279)

	Adenoma (n=71)	Colorectal cancer (n=62)	Normal (n=146)	P
**Age (mean±SD)**	53.21±13.22	57.33±11.04	55.19±16.08	0.246
**Gender**				0.213
Male	32	37	89	
Female	39	25	57	
**pTNM Stages**				
Ⅰ-Ⅱ	-	23	-	
Ⅲ-Ⅳ	-	39	-	
**Polyp size (cm)**				
<0.5	18			
0.5-1	39			
≥1	14			
**Adenoma classification**				
tubular	47			
tubulovillous	14			
villous	11			

**Table 2:** Methylation of targets in colorectal cancer, polyps and normal mucosa (n=279)

	SNCA	SPG20	FBN1	SEPT9
**3 groups**				
Colorectal cancer(Ca)	56/62(90.32%)	53/62(85.48%)	49/62(79.03% )	57/62(91.93%)
Adenoma(A)	33/71(46.47%)	40/71(56.33%)	36/71(50.70%)	40/71(56.33%)
Normal mucosa(N)	21 /146(14.38%)	40/146(27.40%)	19/146(13.01%)	9/146(6.16%)
p	Ca vs N 0.000	Ca vs N 0.000	Ca vs N 0.000	Ca vs N 0.000
	A vs N 0.000	A vs N 0.000	A vs N 0.000	A vs N 0.000
	Ca+A vs N 0.000	Ca+A vs N 0.000	Ca+A vs N 0.000	Ca+A vs N 0.000
	Ca vs A0.000	Ca vs A0.000	Ca vs A0.000	Ca vs A0.000
**Adenoma size**				
<0.5cm	5/18 (27.78%)	8/18 (44.44%)	5/18 (27.78%)	13/18 (72.227%)
0.5-1cm	16/39 (41.02%)	21/39 (53.84%)	23/39 (58.97%)	27/39 (69.23%)
≥1cm	12/14 (85.72%)	11/14 (78.57%)	8/14 (57.14%)	10/14 (71.43%)
P	0.033	0.112	0.060	0.935
**Adenoma classification**				
tubular	21/47	23/47	22/47	24/47
tubulovillous	7/14	8/14	9/14	7/14
villous	5/11	9/11	5/11	9/11
p	0.915	0.071	0.866	0.056
**pTNM Stages**				
Ⅰ-Ⅱ	20/23(86.95%)	19/23(82.61%)	18/23(78.26%)	22/23(95.654%)
Ⅲ-Ⅳ	36/39(92.30%)	34/39(87.18%)	31/39(79.49%)	35/39(89.74%)
P	0.495	0.624	0.615	0.413

**Table 3:** Screening value for colorectal cancer and adenoma when one molecular marker was positive (n=279)

Gene	Se %	Sp %	PPV %	NPV %	AUC	95% CI	P
**Cancer**							
SNCA	80.6	75.2	61.7	88.6	0.779	0.712-0.846	0.000
SPG20	76.4	68.3	54.5	85.3	0.723	0.651-0.795	0.000
FBN1	75.0	82.8	68.4	87.0	0.789	0.721-0.857	0.000
SEPT9	90.3	93.4	85.5	95.0	0.913	0.867-0.960	0.000
**Adenoma**							
SNCA	41.0	77.2	41.0	75.2	0.581	0.493-0.668	0.047
SPG20	52.5	72.6	41.2	77.3	0.604	0.518-0.690	0.019
FBN1	44.3	82.8	51.9	77.9	0.635	0.548-0.722	0.002
SEPT9	42.6	99.2	70.3	79.3	0.675	0.588-0.763	0.000
**Cancer + Adenoma**							
SNCA	62.4	75.2	75.4	67.2	0.688	0.625-0.751	0.000
SPG20	65.4	68.1	71.0	66.1	0.668	0.604-0.733	0.000
FBN1	60.9	88.3	80.8	68.1	0.718	0.657-0.780	0.000
SEPT9	68.4	92.4	90.4	75.0	0.804	0.750-0.859	0.000

**Table 4:** Screening value for colorectal cancer and adenoma when two molecular markers were positive (n=279)

Gene	Se%	Sp%	PPV%	NPV%	AUC	95% CI	P
**Cancer**							
SNCA+SPG20	57.9	93.2	84.6	77.5	0.879	0.813-0.945	0.000
SNCA+FBN1	60.5	94.9	88.5	78.9	0.894	0.830-0.958	0.000
SNCA+SEPT9	73.7	98.3	96.6	85.3	0.953	0.908-0.998	0.000
SPG20+FBN1	52.6	96.6	90.9	76.0	0.895	0.834-0.955	0.000
SPG20+SEPT9	68.4	96.6	92.9	82.6	0.941	0.890-0.992	0.000
FBN1+SEPT9	65.8	98.3	96.2	81.7	0.965	0.933-0.997	0.000
**Adenoma**							
SNCA+SPG20	29.5	93.2	63.6	73.3	0.673	0.547-0.798	0.010
SNCA+FBN1	25.9	94.9	70.0	73.7	0.694	0.568-0.821	0.004
SNCA+SEPT9	33.3	98.3	90.0	76.3	0.702	0.570-0.834	0.003
SPG20+FBN1	29.6	96.6	80.0	75.0	0.710	0.586-0.834	0.002
SPG20+SEPT9	40.7	96.6	84.6	78.1	0.705	0.574-0.836	0.002
FBN1+SEPT9	29.6	98.3	88.9	75.3	0.752	0.630-0.874	0.000
**Cancer + Adenoma**							
SNCA+SPG20	44.6	93.2	87.9	60.4	0.790	0.711-0.870	0.000
SNCA+FBN1	46.2	94.9	90.9	61.5	0.811	0.734-0.888	0.000
SNCA+SEPT9	56.9	98.3	97.4	67.4	0.849	0.778-0.920	0.000
SPG20+FBN1	43.1	96.6	93.3	60.6	0.818	0.744-0.892	0.000
SPG20+SEPT9	56.9	96.6	94.9	67.1	0.843	0.771-0.915	0.000
FBN1+SEPT9	50.8	98.3	97.1	64.4	0.877	0.813-0.940	0.000

**Table 5:** Screening value for colorectal cancer and adenoma when three or four molecular markers were positive

Gene	Se%	Sp%	PPV %	NPV %	AUC	95% CI	P
**Cancer**							
SNCA+SPG20+FBN1	36.8	100.0	100.0	71.1	0.949	0.910-0.989	0.000
SNCA+SPG20+SEPT9	52.6	100.0	100.0	76.6	0.972	0.943-1.000	0.000
SNCA+FBN1+SEPT9	52.6	98.3	95.2	76.3	0.981	0.960-1.000	0.000
SPG20+FBN1+SEPT9	44.7	98.3	94.4	73.4	0.979	0.950-1.000	0.000
4 all positive	31.6	100.0	100.0	69.4	0.987	0.965-1.000	0.000
**Adenoma**							
SNCA+SPG20+FBN1	14.8	100.0	100.0	72.0	0.732	0.610-0.853	0.001
SNCA+SPG20+SEPT9	25.9	100.0	100.0	74.7	0.713	0.584-0.842	0.002
SNCA+FBN1+SEPT9	22.2	98.3	85.7	73.4	0.754	0.632-0.876	0.000
SPG20+FBN1+SEPT9	22.2	98.3	85.7	73.4	0.746	0.619-0.873	0.000
4 all positive	14.8	100.0	100.0	72.0	0.754	0.629-0.879	0.000
**Cancer + Adenoma**							
SNCA+SPG20+FBN1	27.7	100.0	100.0	55.7	0.859	0.793-0.925	0.000
SNCA+SPG20+SEPT9	41.5	100.0	100.0	60.8	0.863	0.797-0.929	0.000
SNCA+FBN1+SEPT9	40.0	98.3	96.3	59.8	0.885	0.824-0.946	0.000
SPG20+FBN1+SEPT9	35.4	98.3	95.8	58.0	0.884	0.819-0.948	0.000
4 all positive	24.6	100.0	100.0	54.6	0.888	0.826-0.950	0.000

**Table 6:** Sensitivity and specificity of iFOBT in screening colorectal cancer and adenoma

		Cancer		Adenoma	
	N cases	sensitivity	specificity	sensitivity	specificity
Wong et al.(2003)	250	100%	87%	53%	65%
Medical Advisory Secretariat(2009)	n.a	81%	94%	28%	91%
Tao et al.(2012)	597	65.7%	97%	19.7%	97%
Chen et al.(2013)	610	96%	72%	58%	72%
Wakamura et al. (2015)^7^	919	91%	71%	56%	73%
Rutka et al.(2016)	95	94.7%	72.5%	80%	72.5%
Aniwan et al.(2017)	1479	78%	82%	42%	94%
Chung et al.(2017)	60	84%	55%	n.a	n.a
